# *Lactobacillus reuteri* DSM 17938 Probiotics May Increase CC-Chemokine Receptor 7 Expression in Infants Treated With for Colic

**DOI:** 10.3389/fped.2019.00292

**Published:** 2019-07-16

**Authors:** Francesco Savino, Ilaria Galliano, Andrea Savino, Valentina Daprà, Paola Montanari, Cristina Calvi, Massimiliano Bergallo

**Affiliations:** ^1^Department of Paediatrics, Azienda Ospedaliera Universitaria Città della Salute e della Scienza di Torino, Turin, Italy; ^2^Department of Public Health and Paediatric Sciences, Scuola di Medicina, Università degli Studi di Torino, Turin, Italy

**Keywords:** breastfeeding, CC-chemokine receptor 7, colicky infants, *Lactobacillus reuteri*, Interleukin 10

## Abstract

**Aim:** Studies have shown that *Lactobacilli reute*ri probiotics can affect cells that play a key role in the immune system. This *in vivo* Italian study investigated how *Lactobacillus reuteri* DSM 17938 influenced CC-chemokine receptor 7 (CCR7) and interleukin 10 (IL-10) in breastfed colicky infants.

**Methods:** Our University hospital in Turin recruited 50 healthy outpatients, at a median age of approximately 1 month, from September 2017 to August 2018. They were randomized to daily *Lactobacillus reuteri* DSM17938 (1 × 10^8^ cfu) or a placebo for 28 days from recruitment. We collected peripheral blood and evaluated the expression of CCR7 messenger ribonucleic acid using the real-time TaqMan reverse transcription polymerase chain reaction method at baseline and after the study period.

**Results:** We found increased expression of CC-chemokine receptor 7 in infants treated with the probiotic, but not the controls (*p* < 0.0026). No differences were observed for interleukin 10 after the study period in either group. At baseline, daily crying time was comparable in the probiotic and control groups: 341 (25) vs. 337 (29) min., respectively (*p* = 0.450). After 28 days, daily mean crying time decrease statistically in the probiotic group: 78 (23) vs. 232 (31), respectively (*p* < 0.001).

**Conclusion:** The increase in CC-chemokine receptor 7 might have been a response to probiotic treatment. As a relatively small sample was used to conduct this study, our research needs to be replicated in different settings, and over time, to produce comparable findings.

## Key notes

- *Lactobacilli reuteri* can affect cells that play a key role in the immune system.
- This *in vivo* study investigated how *Lactobacillus reuteri* DSM 17938 influenced CC-chemokine receptor 7 (CCR7) and interleukin 10 (IL-10) in 50 colicky breastfed infants.
- We found increased expression of CC-chemokine receptor 7 in infants randomized to the probiotic, but not placebo, group and no differences in interleukin 10 in either group.

## Introduction

Inflammatory mediators, such as cytokines, chemokines and chemokines receptors, have been linked to immune system alterations and they display a considerable amount of similarity of function in infants (1). However, their precise mechanisms in early life are not fully understood.

Historically, neonates were assumed to be deficient in regulatory T cells and other adaptive immune cells, but further research has been carried out in this area in the last decade (2).

Chemokine receptors are involved in organizing thymic architecture and function and lymph-node homing of naive and regulatory T cells. They also influence inflammation, particularly chemokine receptor type 7 (CCR7), which is essential for the directed migration of adaptive immune cells, and regulatory T cell generation (3). The CCR7 signaling system has been implicated in diverse biological processes, such as lymph node homeostasis, T cell activation, immune tolerance and inflammatory responses (3)

CCR7 is a member of the G protein-coupled receptor family (syn CD197). It is activated by two different ligands, chemokine ligand 19 and chemokine ligand 21, and is involved in the migration, activation and survival of multiple cell types, including dendritic cells, T cells, eosinophils, B cells and endothelial cells (4).

Interleukin-10 (IL-10) is arguably the most potent anti-inflammatory cytokine and it is produced by nearly all of the innate and adaptive immune cells. IL-10 plays an important role in the maintenance of gut homeostasis, due to its anti-inflammatory functions. During an infection, IL-10 inhibits the activity of type 1 T helper cells, natural killer cells, and macrophages, which are required for optimal pathogen clearance, and contribute to tissue damage (5).

In infants, the mucosal immune system is constantly exposed to a wide range of commensal and potentially pathogenic microbial species. Intestinal intraepithelial lymphocytes provide a first line of protection and investigating their role in immunity is critical (6).

Emerging evidence supports the concept that infant colic could represent gut inflammation and microbial dysbiosis (7, 8). Lactobacilli are widely used as probiotics and have been shown to have beneficial effects on diarrhea that is associated with infections (9). However, they have also been used in clinical trials that have examined necrotising enterocolitis (10), inflammatory bowel diseases, and infantile colic (11).

Probiotics provide beneficial bacteria and clinicians use them because they can improve modifications in the intestinal microbiota and influence the immunological status of the host (9).

The beneficial effect of probiotics are mediated by several mechanisms, such as immune receptor cascade signaling and cytokines and toll like receptors (10) modulating inflammation and gene networks regulating production of bacterial-derived immunoregulatory molecules (12).

In a study published in 2018 we reported that infants with colic who were treated with *Lactobacilli reuteri* DSM17938 for 30 days demonstrated a significant decrease in calprotectin values and crying time. The infants also showed increased forkhead box P3 concentrations and these resulted in a decreased retinoic acid-related orphan receptor T and forkhead box P3 ratio (13), while the expression of mRNA expression of Toll like receptor 2 and Toll like receptor 4 seem not influenced (14). An *in vitro* study Cervantes -Barragan reported that molecular mechanisms responded to the inflammatory status of the gut during treatment with *Lactobacillus reuteri* (15).

One of these regulatory molecules is the IL-10, which is an anti-inflammatory cytokine and its effects have been demonstrated in studies in both mice and humans (14). Indeed, IL-10 signaling is essential for intestinal homeostasis, particularly in macrophages. IL-10 dampens intestinal inflammation and it would be interesting to know if it can be modulated by probiotic treatment (16, 17).

The aim of the present study was to investigate CCR7 and IL-10 in breastfed colicky infants treated with *Lactobacillus reuteri* DSM 17938 for 28 days.

## Patients and Methods

### Subjects and Methods

This study was carried out at the Department of Pediatrics, Regina Margherita Children Hospital, Turin, Italy, between September 2017 and August 2018. We recruited 50 full-term exclusively breastfed infants aged <50 days and randomized equal numbers to receive either *Lactobacillus reuteri* DSM 17938 of a placebo for 28 days. The median age of the probiotic group at recruitment was 28.5 days and it was 32.5 days for the placebo group. They were included if they were born at a gestational age of between 37 and 40 weeks, with a birth weight of between 2,500 and 4,000 g and a 5-min Apgar score of more than seven. All the infants had colic and underwent blood tests during routine outpatient examinations. We used the modified Wessel's criteria for the diagnosis of infantile colic (13).

The study protocol was approved by the local Ethical Committee at Ospedale Mauriziano—Ospedale Infantile Regina Margherita—S. Anna Torino, and the infants' parents provided written consent to participate in the study.

### Sample Collection

Venous blood samples were collected from the infants at 8 a.m., after a 3 h fasting period, which coincided with routine clinical blood sampling to minimize the disturbance to the infants. A tube of haemachrome was collected at recruitment, namely day 1 of the study period, for each infant and at the control visit after 28 days. Each sample was transferred to sterile Eppendorf tubes (Merck KGaA, Darmstadt, Germany) and stored in a freezer at −80°C until they were needed.

### Intervention

The infants were randomized to receive the probiotic *Lactobacilli reuteri* DSM 17938 or placebo daily for 28 days using a computer programme. The active study product consisted of a suspension of freeze-dried *Lactobacilli reuteri* DSM 17938 in a mixture of sunflower oil and a medium-chain triglyceride oil, which was supplied in a 5 ml dark bottle fitted with a dropper cap. Five drops of the formulation delivered the daily dose of *Lactobacilli reuteri* 1 × 10^8^ colony forming units and this was given 30 min before feeding. Both the *Lactobacilli reuteri* and placebo study products were provided by the Italian distributor (Nòos Srl, Roma, Italy). This oil suspension is stable for 21 months at 2°C to 8°C, as documented by the manufacturer (BioGaia AB, Stockholm. Sweden). During the study, parents were instructed to keep the product in the refrigerator when it was not in use.

Subjects received the study product free of charge after enrolment. Each child's pediatrician was informed, in writing, of the child's participation and parents were given a phone number so that they could ask the research team questions and report any perceived problems or concerns during the study.

### Follow-Up Phase

Each participant was given a physical examination at enrolment, on day 1 and on day 28.

The parents completed a daily questionnaire for the duration of the study, which included the following data on the general health status of the infant, namely the occurrence of any illness and the use of antibiotics or other medication. It also included gastrointestinal signs and related symptoms, such as crying time, vomiting and regurgitation, and stooling habits.

**Figure 1:**
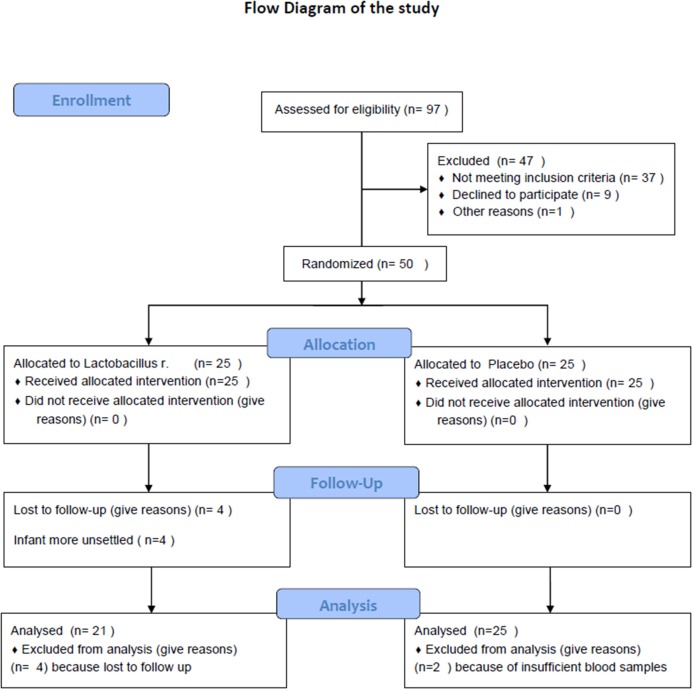
Flow diagram.

Participants flow through study are reported in Figure 1.

### Ribonucleic Acid (RNA) Extraction

Total RNA was extracted from 200 μl of blood using a Maxwell automated extractor (Promega, Wisconsin, USA) and the simplyRNA Blood Kit protocol (Promega) without modification. One microgram of total RNA was reverse-transcribed with 8 μl of 10X buffer, 4.8 μl of 25 mM MgCl_2_, 2 μl of ImpromII (Promega), 1 μl of 40 U/l RNase inhibitor, 0.4 μl of 250 μM random hexamers (Promega), 2 μl of dNTP mix at 100 mM each (Promega) and double distilled water to create a final volume of 20 μl. The reaction was carried out using a GeneAmp PCR system 9700 Thermal Cycler (Applied Biosystems, California, USA) using the following conditions: 5 min at 25°C, 60 min at 42°C and 15 min at 70°C for enzyme inactivation. The complementary deoxyribonucleic acid (DNA) was stored at −80° until use.

### Relative Quantification by Real-Time Polymerase Chain Reaction (PCR)

Relative quantification of the messenger RNA expression levels of the selected genes was achieved using TaqMan amplification (Lifetechnologies, Texas, USA) and normalization to glyceraldehyde-3-phosphate dehydrogenase (GAPDH) as the reference gene. This was achieved using an ABI PRISM 7500 real-time system (Life Technologies, Texas, USA). The mRNA messenger RNA expression levels of CC R7 and IL10 were quantified using real-time PCR. Approximately 100 ng of cDNAcomplementary DNA was amplified in a total volume of 20 μl containing 2 × GoTaq® qPCR Master Mix (Promega, Madison, WI), 500 nmol of specific primers and 200 nmol of probe. Were used: CCR7 primers (CCR7F-5′- GCAACTCAACATCGCCTACG−3′)(CCR7R-5′- GAAGAGATCGTTGCGGAACTT−3′) and probe (CCR7P-6FAM- accctttcttgtacgccttcatcgg -TAMRA); IL10 primers (IL10F-5′- ATGAAGGATCAGCTGGACAACTT−3′) (IL10R-5′- CCTTGATGTCTGGGTCTTGGT-3′) and probe (IL10P-6FAM- ACCTGGGTTGCCAAGCCTTGTCTG -TAMRA); GAPDH primers (GAPDHF-5′-CCAAGGTCATCCATGACAAC-3′) (GAPDHR-5′- GTGGCAGTGATGGCATGGAC-3′) and probe (GAPDH-6FAM- TGGTATCGTGGAAGGA-3′ MGB). The amplifications were performed in a 96-well plate at 95°C for 2 min, followed by 40 cycles of 95°C for 15 s and 60°C for 1 min. Each sample was run in triplicate. The target gene relative expression in patients was compared with normal samples using the 2-ΔΔCt method, and the relative expression is expressed in arbitrary units (AU).

The messenger RNA expression levels of CCR7 and IL10 and GAPDH were quantified by real-time PCR, as previously described by Mareschi et al. (18).

### Statistical Analysis

The sample size was calculated on the basis that we wanted to find a 50 min reduction in daily average crying time in the probiotic group, when it was compared to the placebo group. This was considered a clinically relevant difference and was based on previous studies. We calculated that 20 patients were needed in each group, based on an alpha value of 0.05, beta value of 0.20 and an estimated standard deviation (SD) within the groups of 50 min crying time. We decided to enroll 25 subjects per group to allow for a 20% drop-out rate.

Randomization was performed by the random-digit method, using computer-generated numbers. We used a two-treatment randomization scheme with random blocks of varying size, employing Stata Statistical Software, Release 9 (StataCorp LP, College Station, Texas, USA) and the ralloc procedure. Data were analyzed by SPSS, version 16 (SPSS Inc, Illinois, USA), while the sample size calculation was performed by NCSS-PASS 2000 (Number Cruncher Statistical Systems, Utah, USA).

Data are shown as means and standard deviations (SD) or medians and interquartile ranges (IQR) for continuous variables, as appropriate, and numbers and percentages for categorical variables. Differences between the groups were evaluated using the Student's *t*-test for paired samples, while associations between the categorical variables were evaluated by Fisher's exact test. The Wilcoxon signed-rank test and Friedman test were used to evaluate differences between paired samples for continuous variables, when appropriate.

All reported *p*-values were two-sided and differences were considered to be significant when *p* < 0.05.

## Results

A total of 50 infants were enrolled to the study and allocated, according to the protocol, to receive the *Lactobacilli reuteri* DSM17938 probiotic or placebo. The characteristics of the subjects enrolled and treated with the probiotic and the placebo are reported in Table 1. There were no significant differences between groups at baseline (Table 1). We excluded six infants from the analysis because of sufficient blood samples: four in the probiotic group and two in the placebo group.

**Table 1:** Baseline characteristics of the participants in the two study groups.

**Variables**	**Probiotic (*n* = 25)**	**Placebo (*n* = 25)**	***p*-value**
Type of delivery (Cesarean), *n* (%)	6 (25)	13 (52)	0.079^#^
Male, *n* (%)	14 (56)	15 (60)	1.000^#^
Age at entry, median days (interquartile range)	28.5 (21)	32.5 (21)	0.382^¶^
Family history of GI diseases (yes), *n* (%)	8 (32)	6 (24)	0.754^#^
Family history of atopy (yes), *n* (%)	9 (36)	12 (48)	0.567^#^
Gestational age (weeks)	37.8 ± 0.5	37.9 ± 0.4	0.286^*^

*Data are presented as means and standard deviations (SD)*.

# *Fisher's exact test*;

¶ *Mann-Whitney test*;

* *Student's t-test*.

The primary outcome was the measurement of the expression level of CC-chemokine receptor 7 messenger RNA in the study group at day 1 and at day 28.

The CC-chemokine receptor 7 values in the probiotic group on day 1 was 2.73 ± 1.1 ΔCt and on day 28 it was 1.82±1.45 ΔCt. In comparison, the CC-chemokine receptor 7 values in the placebo group was 1.86±1.1 ΔCt on day 1 and 1.21±0.6 ΔCt on day 28 (Table 2).

**Table 2:** Blood inflammatory markers (ΔCt), in infant with colic at enrolment and after 28 days of supplementation with *L. reuteri* or Placebo.

**Variables**	**Probiotic (*n =* 25)**	**Placebo (*n =* 25)**	***p*-value**
IL 10 (ΔCt) DS (day 1)	8.64 ± 1.10	8.61 ± 1.40	0.111^¶^
IL 10 (ΔCt) DS (day 28)	8.84 ± 2.31	9.31 ± 1.30	
CCR7 (ΔCt) DS (day 1)	2.73 ± 1.10	1.86 ± 1.10	0.86^¶^
CCR7 (ΔCt) DS (day 28)	1.82 ± 1.45	1.21 ± 0.60	

*Data are presented as means and standard deviations (SD)*.

¶ *Mann-Whitney test*.

**Figure 2:**
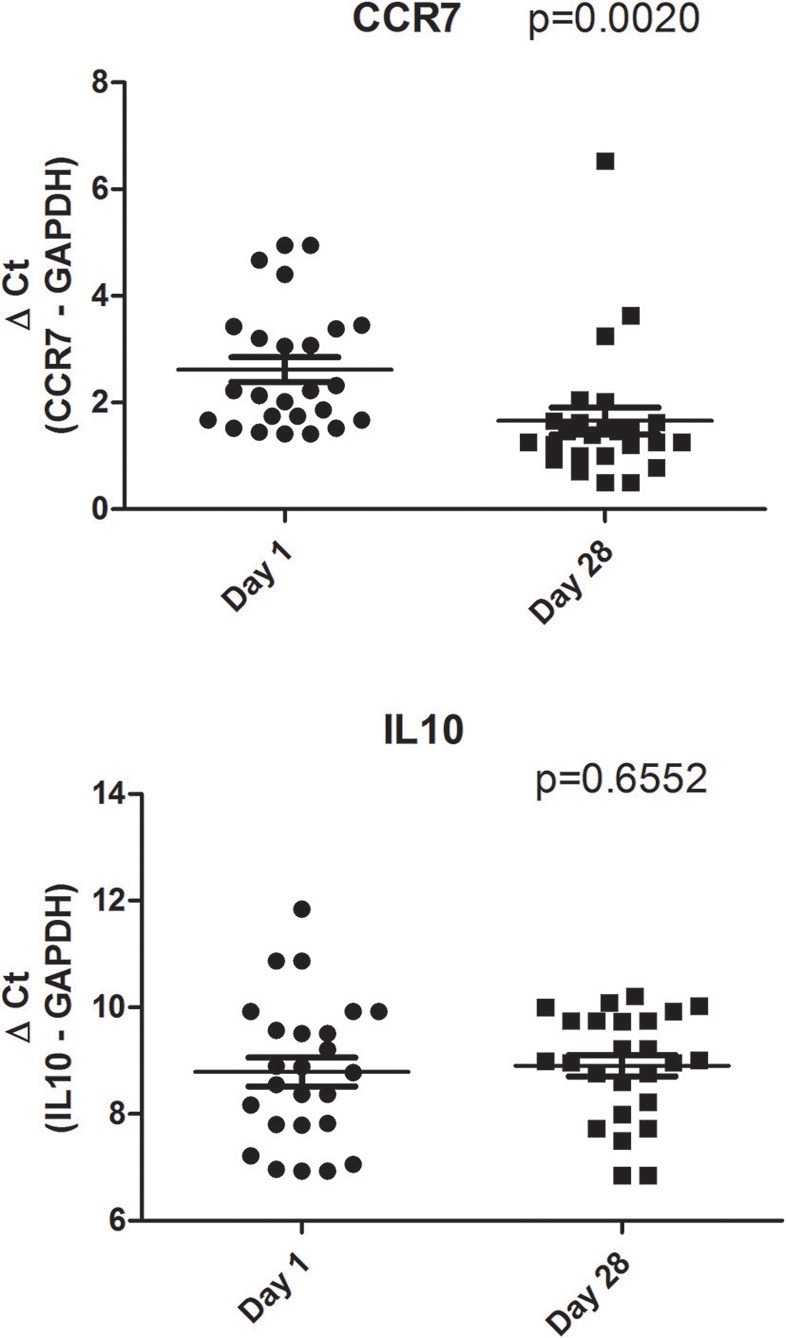
CCR7 and IL10 transcriptional levels in the venous blood of group treated with L Reuteri DSM 17938. Data are represented as circle plots (day 1) and box plots (day 28), with horizontal lines depicting the averages. Relative messenger RNA levels were analyzed by real-time PCR and represented by ΔCt. The Paired *t*-test was performed.

After the study period CC-chemokine receptor 7 were expressed at higher levels in the probiotic group than in the placebo group (*p* = 0.0020) (Figure 2).

When we analyzed the placebo group we did not observe different CCR7 expression on day 1 and on day 28 (*p* = 0.26) (data not shown).

### Secondary Outcome

The values of IL-10 in the probiotic group were 8.64 ± 1.32 ΔCt on day 1, and 8.84 ± 2.31 ΔCt on day 28 (*p* = 0.6552)(Figure 2). The values in the placebo group were 8.61 ± 1.40 ΔCt on day 1 and 9.31 ± 1.30 ΔCt on day 28 (*p* = 0.62) (data not shown). At day 28, IL10 was equally expressed in the probiotic (Figure 2) and control groups (*p* = 0.4375) (data not shown).

Crying time of infants treated with *L. reuteri* or Placebo are reported in Table 3.

**Table 3:** Crying and fussing time (mean minutes per days) at day 1 and at day 28 in placebo and *L. reuteri* group.

**Variable (minutes)**	**Placebo (*n* = 25)**	***L. reuteri* (*n* = 25)**	***P*-value**
Crying time per day 1	337.21 ± 29.2	341.55 ± 25.80	N.S.
Crying time per day 28	232.24 ± 31	78.34 ± 23.24	<0.001^¶^

*Data as mean ± standard deviation*.

Statistical analysis:

¶ *Mann-Whitney test*.

After *L. reuteri* administration for 28 days in infants with colic, we observed a significant decrease of daily crying time (341.55 ± 25.80 min/day at day 0 vs. 78.34 ± 23.24 min/day at day 28, *p* = 0.001) (Table 3).

We conducted on response variable (crying time) an ITT (Intention to Treat) analysis too, including 4 infants dropped out. As they were all from the Placebo group we classified them as responders.

Infants Responders (with a reduction of 50% in crying time from baseline) were significantly higher in the *L. reuteri* group vs. placebo group on days 28 (21 vs. 11, *p* = 0.007).

## Discussion

The role of the main cytokines, chemokines and their receptors in the pathophysiology of disorders that involve inflammation in an emerging issue of active research (15, 16, 19–22).

Gut microbiota and its metabolites have been shown to influence immune functions and immune homeostasis within the gut, particularly during the first few months of life. In addition, probiotics have been shown to have an effect on immunity throughout cytokines and the production of metabolites such as tryptophan (15). Haileselassie et al. reported that *Lactobacillus reut*eri was able to influence the generation of monocyte-derived dendritic cells and subsequent autologous T cell responses (23).

On the other hand an *in vitro* study by Cervantes et al. demonstrated that *Lactobacillus reuteri* induced gut intraepithelial CD4(+)CD8αα(+) T cells (15).

The immunological mechanism behind the effects of probiotic such as *Lactobacillus reuteri* are probably regulatory T cells cell, as previous studies have reported (13, 14, 22, 23).

In fact Yuying et al. showed that *Lactobacillus reuteri* DSM 17938 changed the frequency of forkhead box P3 ratio regulatory T cells in the intestine and mesenteric lymph node in experimental necrotising enterocolitis (24).

While the precise mechanism is unclear, we can argue that the reaction between *Lactobacillus reuteri* and both epithelial and non-epithelial enteric cells must be active in modulating intrinsic anti-inflammatory effects in the intestine, as shown in animal models *in vivo* (23–27).

In this study we showed that *Lactobacillus. reuteri* 17938 was effective in alleviation of crying time due to colic in breast fed infants. Our results are consistent with the recent meta-analysis of previous four randomized trials (11).

Our findings showed also an increased expression of CC-chemokine receptor 7 in infants treated with *Lactobacilli reuteri* DSM 17938 after 28 days (p = < 0.0020), compared to day 1, while no differences where observed for IL-10 values.

CCR7 is involved in efficient induction of immune reactions as well as their silencing and regulation. Most of the knowledge on the involvement of CCR7 in the development of immunity and tolerance has been derived from mouse models and data on CCR7 function in humans is rather sparse (3, 4). However, it has been discovered that CCR7 contributes to the induction and maintenance of tolerance and this suggests a possible new strategy for treatment using probiotics.

CCR7 has recently been shown to be up-regulated in peripheral blood DCs and in DCs derived from monocytes and CD34 progenitor cells after activation with a variety of agents (28).

Our *in vivo* results using peripheral blood mononuclear cells from treated infants with L reuteri showed up-regulation of CCR7 expression levels. that the baseline levels of CCR7 were low.

These results, preliminary in nature, are consistent with the hypothesis that level of expression of CCR7 maybe the response to recruitment to gut lymphatics systems.

While our data suggest that IL-10 cytokines seem not be involved by probiotic supplementation, but a larger sample is necessary to confirm our observations, which were echoed by an animal study carried out by Yan et al. (29).

### Strength and Limitations

There were some limitations to our study. First, since this was an experimental study, it only provides preliminary data. It is difficult to obtain detailed information on many factors when carrying out a pilot study with a small number of subjects. That is why it is necessary to control for the effects of extraneous variables that might result in misleading interpretations of causality. As a result, the findings of this study cannot be generalized.

However, to the best of our knowledge this is the first research study that investigated CCR7 in healthy breastfed infants treated with *Lactobacillus reuteri* DSM 17938 compared to a placebo using real time PCR. Tompa et al. reported that cryopreservation of peripheral blood mononuclear cells could have an impact on the expression of CCR7 on regulatory T cells, but we did not use cytofluorimetry and our data were not influenced by storage (30).

The standardized approach that we used permits the study to be replicated in different settings, or over time, to produce comparable findings.

## Conclusion

Our randomized study of breastfed infants with colic found a decreased crying time and an increased expression of CC-chemokine receptor 7 in infants treated with the probiotic *Lactobacillus reuteri* DSM 17938 for 28 days, but not the placebo group. No differences were observed for interleukin 10 after the study period in either group. The increase in CC-chemokine receptor 7 might have been a response to probiotic treatment.

Understanding the possible effect of probiotic supplements, particularly during the first few weeks of life, is interesting and could also help to induce oral tolerance. Future studies on CCR7 in human molecular medicine, as well as more refined microbiota models of site-specific and inducible CCR7 and the CCR7-ligand, will help our understanding of this important molecular pathway. It will also aid our understanding of the cellular responses driven by it during probiotic treatment. Taking advantage of these new features may lead to a tailored approach for regulatory T cells using probiotic treatment.

## Data Availability

The raw data supporting the conclusions of this manuscript will be made available by the authors, without undue reservation, to any qualified researcher.

## Ethics Statement

The study protocol was approved by the local Ethical Committee at Ospedale Mauriziano—Ospedale Infantile Regina Margherita–S. Anna Torino, and the infants' parents provided written consent to participate in the study.

## Author Contributions

FS had primary responsibility for protocol development as principal investigator and wrote the manuscript. IG performed PCR analysis and helped to write the manuscript. AS analyzed data and wrote the manuscript, edited references. VD performed PCR analysis and helped to write the manuscript. PM performed PCR analysis and helped to write the manuscript. CC performed analysis and wrote the manuscript. MB performed the final data analysis, supervised analysis and helped to write the manuscript.

### Conflict of Interest Statement

The authors declare that the research was conducted in the absence of any commercial or financial relationships that could be construed as a potential conflict of interest.

## Abbreviations

CCR7: chemokine receptor type 7
IL10: interleukin 10
GAPDH: glyceraldehyde 3-phosphate dehydrogenase
DNA: deoxyribonucleic acid
RNA: ribonucleic acid
PCR: polymerase chain reaction.

## References

[1] Basha S Surendran N Pichichero M. Immune responses in neonates. Expert Rev Clin Immunol. (2014) 10:1171–84. 10.1586/1744666X.2014.942288 25088080 PMC4407563

[2] Adkins B Leclerc C Marshall-clarke S. neonatal adaptive immunity comes of age. Nat Rev Immunol. (2004) 4:553–64. 10.1038/nri1394 15229474

[3] Förster R Davalos-Misslitz AC Rot A. CCR7 and its ligands: balancing immunity and tolerance. Nat Rev Immunol. (2008) 8:362–71. 10.1038/nri2297 18379575

[4] Raju R Gadakh S Gopal P George B Advani J Soman S. Differential ligand-signaling network of CCL19/CCL21-CCR7 system. Database. (2015) 2015:bav106. 10.1093/database/bav106 26504105 PMC4620938

[5] Couper KN Blount DG Riley EM. IL-10: the master regulator of immunity to infection. J Immunol. (2008) 180:5771–7. 10.4049/jimmunol.180.9.5771 18424693

[6] Sheridan BS Lefrançois L. Intraepithelial lymphocytes: to serve and protect. Curr Gastroenterol Rep. (2010) 12:513–21. 10.1007/s11894-010-0148-6 20890736 PMC3224371

[7] Mai T Fatheree NY Gleason W Liu Y Rhoads JM. Infantile colic: new insights into an old problem. Gastroenterol Clin North Am. (2018) 47:829–44. 10.1016/j.gtc.2018.07.008 30337035 PMC6659398

[8] Rhoads JM Collins J Fatheree NY Hashmi SS Taylor CM Luo M. Infant colic represents gut inflammation and dysbiosis. J Pediatr. (2018) 203:55–61. 10.1016/j.jpeds.2018.07.042 30177353 PMC6669027

[9] Plaza-Díaz J Ruiz-Ojeda FJ Gil-Campos M Gil A. Immune-mediated mechanisms of action of probiotics and synbiotics in treating pediatric intestinal diseases. Nutrients. (2018) 10:E42. 10.3390/nu10010042 29303974 PMC5793270

[10] Hoang TK He B Wang T Tran DQ Rhoads JM Liu Y. Protective effect of *Lactobacillus reuteri* DSM 17938 against experimental necrotizing enterocolitis is mediated by Toll-like receptor 2. Am J Physiol Gastrointest Liver Physiol. (2018) 315:G231–40. 10.1152/ajpgi.00084.2017 29648878 PMC6139641

[11] Sung V D'Amico F Cabana MD Chau K Koren G Savino F. *Lactobacillus reuteri* to treat infant colic: a meta-analysis. Pediatrics. (2018) 141:e20171811. 10.1542/peds.2017-1811 29279326

[12] Thomas CM Saulnier DM Spinler JK Hemarajata P Gao C Jones SE Fol C2-mediated folate metabolism contributes to suppression of inflammation by probiotic *Lactobacillus reuteri*. Microbiol Open. (2016) 5:802–18. 10.1002/mbo3.371 PMC5061717 27353144

[13] Savino F Garro M Montanari P Galliano I Bergallo M. Crying time and RORγ/FOXP3 expression in *Lactobacillus reuteri* DSM17938-treated infants with colic: a randomized trial. J Pediatr. (2018) 192:171–7.e1. 10.1016/j.jpeds.2017.08.062 28969887

[14] Savino F Galliano I Garro M Savino A Daprà V Montanari P Regulatory T cells and Toll-like receptor 2 and 4 mRNA expression in infants with colic treated with *Lactobacillus reuteri* DSM17938. Benef Microbes. (2018) 8:1–10. 10.3920/BM2017.0194 30406696

[15] Cervantes-Barragan L Chai JN Tianero MD Di Luccia B Ahern PP Merriman J. *Lactobacillus reuteri* induces gut intraepithelial CD4(+)CD8αα(+) T cells. Science. (2017) 357:806–10. 10.1126/science.aah5825 28775213 PMC5687812

[16] Cervantes-Barragan L Colonna M. Chemical sensing in development and function of intestinal lymphocytes. Curr Opin Immunol. (2018) 50:112–6. 10.1016/j.coi.2018.01.004 29452963 PMC7456567

[17] Engelhardt KR Grimbacher B. IL-10 in humans: lessons from the gut, IL-10/IL-10 receptor deficiencies, and IL-10 polymorphisms. Curr Top Microbiol Immunol. (2014) 380:1–18. 10.1007/978-3-662-43492-5_1 25004811

[18] Mareschi K Castiglia S Sanavio F Rustichelli D Muraro M Defedele D. Immunoregulatory effects on T lymphocytes by human mesenchymal stromal cells isolated from bone marrow, amniotic fluid, and placenta. Exp Hematol. (2016) 44:138–50.e1. 10.1016/j.exphem.2015.10.009 26577566

[19] Turner MD Nedjai B Hurst T Pennington DJ. Cytokines and chemokines: at the crossroads of cell signalling and inflammatory disease. Biochim Biophys Acta. (2014) 1843:2563–82. 10.1016/j.bbamcr.2014.05.014 24892271

[20] Dinan TG Cryan JF. Regulation of the stress response by the gut microbiota: implications for psychoneuroendocrinology. Psychoneuroendocrinology. (2012) 37:1369–78. 10.1016/j.psyneuen.2012.03.007 22483040

[21] Gensollen T Iyer SS Kasper DL Blumberg RS. How colonization by microbiota in early life shapes the immune system. Science. (2016) 352:539–44. 10.1126/science.aad9378 27126036 PMC5050524

[22] Liu Y Fatheree NY Mangalat N Rhoads JM. Human-derived probiotic *Lactobacillus reuteri* strains differentially reduce intestinal inflammation. Am J Physiol Gastrointest Liver Physiol. (2010) 299:1087–96. 10.1152/ajpgi.00124.2010 20798357 PMC2993169

[23] Haileselassie Y Navis M Vu N Qazi KR Rethi B Sverremark-Ekström E. *Lactobacillus reuteri* and Staphylococcus aureus differentially influence the generation of monocyte-derived dendritic cells and subsequent autologous T cell responses. Immun Inflamm Dis. (2016) 4:315–26. 10.1002/iid3.115 27621814 PMC5004286

[24] Liu Y Fatheree NY Dingle BM Tran DQ Rhoads JM. *Lactobacillus reuteri* DSM 17938 changes the frequency of Foxp3+ regulatory T cells in the intestine and mesenteric lymph node in experimental necrotizing enterocolitis. PLoS ONE. (2013) 8:e56547. 10.1371/journal.pone.0056547 23437165 PMC3577854

[25] Round JL Mazmanian SK. Inducible Foxp3+ regulatory T-cell development by a commensal bacterium of the intestinal microbiota. Proc Natl Acad Sci USA. (2010) 107:12204–9. 10.1073/pnas.0909122107 20566854 PMC2901479

[26] Taylor A Hale J Wiltschut J Lehmann H Dunstan JA Prescott SL. Evaluation of the effects of probiotic supplementation from the neonatal period on innate immune development in infancy. Clin Exp Allergy. (2006) 36:1218–26. 10.1111/j.1365-2222.2006.02552.x 17014428

[27] He B Hoang TK Wang T Ferris M Taylor CM Tian X. Resetting microbiota by *Lactobacillus reuteri* inhibits T reg deficiency–induced autoimmunity via adenosine A2A receptors. J Exp Med. (2017) 214:107–23. 10.1084/jem.20160961 27994068 PMC5206500

[28] Saeki H Moore AM Brown MJ Hwang ST. Cutting edge: secondary lymphoid-tissue chemokine (SLC) and CC chemokine receptor 7 (CCR7) participate in the emigration pathway of mature dendritic cells from theskin to regional lymph nodes. J Immunol. (1999) 162:2472–5. 10072485

[29] Yan FF Murugesan GR Cheng HW. Effects of probiotic supplementation on performance traits, bone mineralization, cecal microbial composition, cytokines and corticosterone in laying hens. Animal. (2019) 13:33–41. 10.1017/S175173111800109X 29785889

[30] Tompa A Nilsson-Bowers A Faresjö M Subsets of CD4+, CD8+, and CD25hi lymphocytes are in general not influenced by isolation and long-term cryopreservation. J Immunol. (2018) 201:1799–809. 10.4049/jimmunol.1701409 30082322

